# Supplementary material for: The incidence, characteristics, impact and risk factors of post-COVID chronic pain in Thailand: A single-center cross-sectional study
Source: PLoS One. 2024 Jan 12;19(1):e0296700. doi: 10.1371/journal.pone.0296700 (PMC10786369; doi:10.1371/journal.pone.0296700)
Supplement: S2 Appendix — (PDF) [file pone.0296700.s004.pdf]

บทสนทนาทางโทรศัพท์

ชื่อโครงการวิจัย อุบัติการณ์ของความปวดเรื้อรังและผลกระทบหลังจากติดเชื้อ COVID-19 ในประเทศไทย

ผู้สัมภาษณ์: สวัสดีค่ะ/ครับ เรียนสาย

คุณ____

ดิฉัน/กระผม ชื่อ______________________โทรมาจากโรงพยาบาลศิริราช ขออนุญาตโทรมาสอบถามอาการหลังจากติดเชื้อโคโรนาไวรัส 2019 ของคุณ______________ค่ะ/ครับ จะขออนุญาตชี้แจงรายละเอียดเกี่ยวกับโครงการวิจัยข้างต้นก่อนนะคะ/ครับ

ชื่อโครงการวิจัย อุบัติการณ์ของความปวดเรื้อรังหลังจากติดเชื้อ COVID-19 และผลกระทบในประเทศไทย

ชื่อหัวหน้าโครงการวิจัย รศ. นพ.นันตสรณ์ สิญจน์บุณยะกุล ภาควิชาวิสัญญีวิทยา คณะแพทยศาสตร์ศิริราชพยาบาล

ที่มาและความสำคัญ

เนื่องจากการระบาดของเชื้อไวรัสโคโรนา 2019 ส่งผลต่อการดำรงชีวิตของมนุษย์ทั่วโลก ทั้งการเจ็บป่วยทางร่างกาย ส่งผลกระทบต่อสภาพอารมณ์ สังคม และการดำเนินชีวิตในระยะฉับพลัน อย่างไรก็ตามมีการรายงานถึงปัญหาในระยะยาวและภาวะแทรกซ้อนหลังจากการติดเชื้อไวรัสโคโรนา 2019 ผู้ป่วยอาจมีภาวะแทรกซ้อนที่เกิดขึ้นหลังการติดเชื้อ ได้แก่ อาการทางปอดเรื้อรัง อาการทางระบบประสาทเรื้อรัง และอาการที่พบทั่วไป เช่น อ่อนเพลียเรื้อรัง หายใจเหนื่อยและสั้น ปวดศีรษะ เจ็บหน้าอก ปวดกล้ามเนื้อละข้อ ตามมาด้วย ซึมเศร้า วิตกกังวล นอนไม่หลับ และสมองล้า มากกว่านั้นมีรายงานว่า ผู้ป่วยที่ติดเชื้อไวรัสโคโรนา 2019 มีอุบัติการณ์ของความปวดเรื้อรังมากกว่าผู้ป่วยที่มานอนโรงพยาบาลในช่วงเวลาเดียวกัน ทางทีมผู้วิจัยเล็งเห็นความสำคัญของการดูแลผู้ป่วยในระยะยาวโดยเฉพาะอย่างยิ่งความปวดเรื้อรังและคุณภาพชีวิต ซึ่งเป็นผลกระทบจากการติดเชื้อไวรัสโคโรนา 2019 จึงขอเรียนเชิญคุณ____________เข้าร่วมวิจัยนี้ค่ะ/ครับ

วัตถุประสงค์หลัก

เพื่อหาอุบัติการณ์เกิดความปวดเรื้อรังและอาการปวดที่เกิดขึ้นใหม่หลังจากติดเชื้อโคโรนาไวรัส 2019 ที่ 3 6 และ 12 เดือนหลังการติดเชื้อในประชากรไทยโรงพยาบาลศิริราช

จำนวนผู้เข้าร่วมโครงการวิจัยทั้งหมด 1,000 ราย

เนื่องจากคนไข้ได้รับการวินิจฉัยติดเชื้อโคโรนาไวรัส 2019 ในช่วงเวลาที่ผ่านมา จึงขออนุญาตสอบถาม และเก็บข้อมูลสำหรับโครงการวิจัยดังกล่าวค่ะ/ครับ

หากคุณมีอาการปวดเรื้อรังคุณจะถูกโทรศัพท์เพื่อสัมภาษณ์เพิ่มเติมอีก 2 ครั้ง ซึ่งจะใช้เวลาประมาณ 5 นาทีต่อครั้ง รวมถึงการสัมภาษณ์ในครั้งนี้ด้วย 10 นาที รวมเป็นเวลาทั้งหมด 20 นาที แต่หากคุณไม่มีความปวดเรื้อรังคุณจะได้รับการสัมภาษณ์ครั้งนี้ครั้งเดียวซึ่งจะใช้เวลาประมาณ 10 นาทีค่ะ/ครับ

ข้อมูลที่สัมภาษณ์จะเป็นข้อมูลของคุณ_____และข้อมูลทั่วไปที่เกี่ยวข้องกับการติดเชื้อไวรัสโคโรนา 2019 ได้แก่ ประวัติการฉีดวัคซีน โรคประจำตัว ประวัติความปวดเรื้อรังเดิม สภาพอารมณ์ อาการที่เกี่ยวข้องกับการติดเชื้อ และแบบสอบถามเกี่ยวกับคุณภาพชีวิตหากไม่มีความปวดเรื้อรัง และแบบสอบถามเกี่ยวกับความปวดร่วมด้วยหากมีความปวดเรื้อรัง คุณ_____อนุญาตหรือไม่คะ/ครับ

ผู้ป่วย: อนุญาต ค่ะ/ครับ

หรือ ไม่อนุญาต ค่ะ/ครับ

(ถ้าผู้ป่วยอนุญาตให้ทีมวิจัยสอบถาม และเก็บข้อมูลสำหรับโครงการวิจัย จะดำเนินการถามแบบสอบถามส่วนถัดไป)

คำถามส่วนที่ 1 ข้อมูลทั่วไป

ผู้สัมภาษณ์: คนไข้อายุเท่าไร คะ/ครับ

ผู้ป่วย: อายุ___________ปี ค่ะ/ครับ

ผู้สัมภาษณ์: คนไข้มีโรคประจำตัวไหม คะ/ครับ

ผู้ป่วย: ไม่มีโรคประจำตัว หรือ มีโรคประจำตัว คือ____________________ค่ะ/ครับ

ผู้สัมภาษณ์: สภาพอารมณ์ในปัจจุบัน เป็นอย่างไรบ้าง คะ/ครับ (ปกติ มีเบื่อหน่าย ท้อแท้ หงุดหงิด ฉุนเฉียว)

ผู้ป่วย: _______________________ ค่ะ/ครับ หรือ มีอาการ_____________________ค่ะ/ครับ

(ข้อมูลในส่วนของรายละเอียดการติดเชื้อโคโรนาไวรัส 2019 ประวัติการได้รับวัคซีนโคโรน่าไวรัส 2019 และประวัติความปวดเรื้อรังในอดีต จะได้รับการค้นจากเวชระเบียนหลังจากได้รับอนุญาต)

คำถามส่วนที่ 2 ข้อมูลที่เกี่ยวข้องกับการติดเชื้อโคโรน่าไวรัส 2019

ผู้สัมภาษณ์: ขออนุญาตสอบถามข้อมูลที่เกี่ยวข้องกับการติดเชื้อโคโรน่าไวรัส 2019 นะคะ/ครับ ขณะติดเชื้อโคโรน่าไวรัส 2019 คนไข้มีอาการอ่อนล้าหรือไม่ คะ/ครับ

ผู้ป่วย: มี หรือไม่มี ค่ะ/ครับ

ผู้สัมภาษณ์: ขณะติดเชื้อโคโรน่าไวรัส 2019 คนไข้มีอาการจมูกไม่ได้กลิ่น หรือไม่ คะ/ครับ

ผู้ป่วย: มี หรือไม่มี ค่ะ/ครับ

ผู้สัมภาษณ์: ขณะติดเชื้อโคโรน่าไวรัส 2019 คนไข้มีอาการปวด หรือไม่ คะ/ครับ

ผู้ป่วย: มี หรือไม่มี ค่ะ/ครับ

ผู้สัมภาษณ์: ความปวดที่เกิดขึ้น เป็นหลังจากได้รับการติดเชื้อโคโรน่าไวรัส 2019 หรือไม่ คะ/ครับ

ผู้ป่วย: ใช่ หรือไม่ใช่ ค่ะ/ครับ

ผู้สัมภาษณ์: ก่อนที่มีการติดเชื้อโคโรน่าไวรัส 2019 คนไข้มีความปวดอยู่แล้ว หรือไม่ คะ/ครับ

ผู้ป่วย: ใช่ หรือไม่ใช่ ค่ะ/ครับ

เมื่อมีการสัมภาษณ์ถึงส่วนนี้ ผู้ป่วยจะถูกแบ่งออกเป็น 4 กลุ่ม ได้แก่

i. กลุ่มที่ 1 ไม่มีความปวด

ii. กลุ่มที่ 2 มีความปวดหลังจากติดเชื้อโคโรน่าไวรัส 2019 (ความปวดที่เกิดขึ้นใหม่หลังจากการติดเชื้อ ที่ไม่เกี่ยวข้องกับภาวะแทรกซ้อนจากการรักษา หรือ ความปวดเรื้อรังเดิม หรือ ความปวดอื่นๆ ที่สามารถมีสาเหตุชัดเจน)

iii. กลุ่มที่ 3 มีความปวดเรื้อรังอยู่เดิมและความปวดถูกกระตุ้นจากการติดเชื้อโคโรนาไวรัส 2019

iv. กลุ่มที่ 4 มีความปวดเรื้อรังอยู่เดิมแต่ไม่ถูกกระตุ้นด้วยการติดเชื้อโคโรน่าไวรัส 2019

ถ้าผู้ป่วยมีรายงานความปวดจากกลุ่มที่ 2

ผู้ป่วยจะถูกประเมิน ระยะเวลาลักษณะความปวด ชนิดยาที่ใช้ ปัจจัยที่ทำให้มีความปวด ปัจจัยที่ทำให้ความปวดบรรเทา ความรุนแรงของความปวดด้วยการวัด Numerical Rating Scale: 0-10, 0= ไม่มีความปวด 10=มีความปวดมากที่สุดที่จะสามารถบรรยายได้ ภาวะแทรกซ้อนจากความปวดด้วยแบบสอบถาม Brief Pain Inventory ฉบับภาษาไทย คุณภาพชีวิตด้วยแบบสอบถาม EuroQol 5D5L ฉบับภาษาไทย ที่ 3, 6 และ 12 เดือน หลังจากติดเชื้อ

ถ้าผู้ป่วยไม่มีความปวด หรืออยู่ในกลุ่มที่ 1, 3 และ 4

ผู้ป่วยจะถูกประเมินคุณภาพชีวิตด้วยแบบสอบถาม EuroQol 5D5L ที่ 3 เดือน

Case record form: TIPC study

Participant No: ☐☐☐☐

Severity: Red/Yellow/ Green

Date of confirmed COVID: ☐☐/☐☐/☐☐☐☐

Date of interview: ☐☐/☐☐/☐☐☐☐ by...

1. Demographic data	<u>แบบประเมินคุณภาพชีวิต EuroQol 5D 5L</u>
HN ____________________<br>Sex ☐ Male ☐ Female<br>Age __________ years	**การเคลื่อนไหว**<br>ข้าพเจ้าไม่มีปัญหาในการเดิน ☐<br>ข้าพเจ้ามีปัญหาในการเดินเล็กน้อย ☐<br>ข้าพเจ้ามีปัญหาในการเดินปานกลาง ☐<br>ข้าพเจ้ามีปัญหาในการเดินอย่างมาก ☐<br>ข้าพเจ้าเดินไม่ได้ ☐
**2. Clinical data**	**การดูแลตนเอง**
**- Vaccination status:** ☐ Yes ☐ No<br>If yes: Dose1 Brand____________ Date☐☐/☐☐/☐☐,<br>Dose2 Brand_______________ Date☐☐/☐☐/☐☐,<br>Dose3 Brand_______________ Date☐☐/☐☐/☐☐,<br>Dose4 Brand_______________ Date☐☐/☐☐/☐☐<br>**- History of chronic pain:** ☐ Yes ☐ No<br>If yes please define (ICD11)<br>1) __________2) __________3) __________<br>**- Underlying disease/Comorbidity:** ☐ Yes ☐ No<br>If yes please define 1) ________2) ________3) ________<br>**- Mental health:** ☐ Yes ☐ No<br>If yes please define___________________	ข้าพเจ้าไม่มีปัญหาในการอาบน้ำ หรือใส่เสื้อผ้าด้วยตนเอง ☐<br>ข้าพเจ้ามีปัญหาในการอาบน้ำ หรือใส่เสื้อผ้าด้วยตนเองเล็กน้อย ☐<br>ข้าพเจ้ามีปัญหาในการอาบน้ำ หรือใส่เสื้อผ้าด้วยตนเองปานกลาง ☐<br>ข้าพเจ้ามีปัญหาในการอาบน้ำ หรือใส่เสื้อผ้าด้วยตนเองอย่างมาก ☐<br>ข้าพเจ้าอาบน้ำ หรือใส่เสื้อผ้าด้วยตนเองไม่ได้ ☐<br>**กิจกรรมที่ทำเป็นประจำ** (เช่น ทำงาน, เรียนหนังสือ, ทำงานบ้าน, กิจกรรมในครอบครัว หรือกิจกรรมยามว่าง)<br>ข้าพเจ้าไม่มีปัญหาในการทำกิจกรรมที่ทำเป็นประจำ ☐<br>ข้าพเจ้ามีปัญหาในการทำกิจกรรมที่ทำเป็นประจำเล็กน้อย ☐<br>ข้าพเจ้ามีปัญหาในการทำกิจกรรมที่ทำเป็นประจำปานกลาง ☐<br>ข้าพเจ้ามีปัญหาในการทำกิจกรรมที่ทำเป็นประจำอย่างมาก ☐<br>ข้าพเจ้าทำกิจกรรมที่ทำเป็นประจำไม่ได้ ☐ ☐
3. Symptoms after COVID infection more than 3 months	**อาการเจ็บปวด/อาการไม่สบายตัว**
**Fatigue**: ☐ Yes ☐ No<br>**Anosmia:** ☐ Yes ☐ No<br>**Pain:** ☐ Yes ☐ No<br>If yes for pain, is it a new pain after COVID? ☐Yes ☐No<br>If No (old chronic pain), is it worsen after COVID? ☐Yes ☐No	ข้าพเจ้าไม่มีอาการเจ็บปวดหรืออาการไม่สบายตัว ☐<br>ข้าพเจ้ามีอาการเจ็บปวดหรืออาการไม่สบายตัวเล็กน้อย ☐<br>ข้าพเจ้ามีอาการเจ็บปวดหรืออาการไม่สบายตัวปานกลาง ☐<br>ข้าพเจ้ามีอาการเจ็บปวดหรืออาการไม่สบายตัวอย่างมาก ☐<br>ข้าพเจ้ามีอาการเจ็บปวดหรืออาการไม่สบายตัวอย่างมากที่สุด ☐
After interview: which group do you think the patient is belong to?<br>**Group1** No chronic pain (no, X)<br>**Group2** Post COVID chronic pain (PCCP) (yes, yes, X)<br>**Group3** Chronic pain, aggravated by COVID infection (yes, no, yes)<br>**Group4** Chronic pain, not aggravated by COVID infection (yes, no, no)<br>(It is possible that patient can belong to 2+3 or 2+4 for example new chronic headache (2) + worsen old chronic LBP (3))<br>If the patients have pain that belong to group 2,3 or 4 go to next page**.**	**ความวิตกกังวล/ความซึมเศร้า**<br>ข้าพเจ้าไม่รู้สึกวิตกกังวลหรือซึมเศร้า ☐<br>ข้าพเจ้ารู้สึกวิตกกังวลหรือซึมเศร้าเล็กน้อย ☐<br>ข้าพเจ้ารู้สึกวิตกกังวลหรือซึมเศร้าปานกลาง ☐<br>ข้าพเจ้ารู้สึกวิตกกังวลหรือซึมเศร้าอย่างมาก ☐<br>ข้าพเจ้ารู้สึกวิตกกังวลหรือซึมเศร้าอย่างมากที่สุด ☐

4. Pain Specific questions

แบบสอบถามอย่างสั้นที่ใช้ประเมินผู้ป่วยมะเร็งที่มีอาการปวด Brief Pain Inventory (Short Form)

1. ในชีวิตที่ผ่านมา ท่านคงเคยมีประสบการณ์ของการปวดลักษณะต่างๆ มาบ้างแล้ว เช่น ปวดศีรษะ หรืออาการปวดเคล็ด ขัดยอก ในขณะที่ท่านมีอาการปวดในลักษณะอื่นๆ ที่พิเศษ นอกเหนือไปจากอาการปวดโดยทั่วไปที่ได้กล่าวถึง .

☐ มี ☐ ไม่มี

2. ตามรูปต่อไปนี้ กรุณาระบุตำแหน่งบนร่างกายที่ท่านรู้สึกปวดโดยใช้เครื่องหมายกากบาท (x)

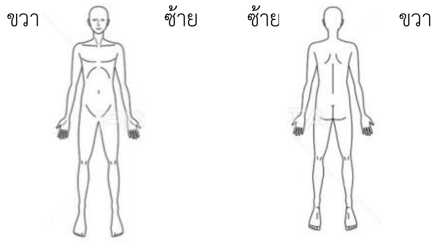

3. กรุณาวงกลมลงบนตัวเลขเพื่อประเมินระดับอาการปวดที่ท่านรู้สึกว่ารุนแรง มากที่สุด ในระยะเวลา 24 ชั่วโมงที่ผ่านมา

0 1 2 3 4 5 6 7 8 9 10

ไม่มีอาการปวด ปวดมากที่สุด

4. กรุณาวงกลมลงบนตัวเลขเพื่อประเมินระดับอาการปวดที่ท่านรู้สึกว่ารุนแรงน้อยที่สุด ในระยะเวลา 24 ชั่วโมงที่ผ่านมา

0 1 2 3 4 5 6 7 8 9 10

ไม่มีอาการปวด ปวดมากที่สุด

5. กรุณาวงกลมลงบนตัวเลขเพื่อประเมินระดับอาการปวด โดยเฉลี่ยของท่าน

0 1 2 3 4 5 6 7 8 9 10

ไม่มีอาการปวด ปวดมากที่สุด

6. กรุณาวงกลมลงบนตัวเลขเพื่อประเมินระดับอาการปวดที่ท่านรู้สึกอยู่ในขณะนี้

0 1 2 3 4 5 6 7 8 9 10

ไม่มีอาการปวด ปวดมากที่สุด

7. ขณะนี้ท่านได้รับการบำบัดรักษาอาการปวดด้วยวิธีใด กรุณาระบุวิธีการรักษา, ชื่อยา และขนาดที่ใช้

**ยาหรือวิธีการรักษาที่ได้รับอยู่ในขณะนี้**	**วัตถุประสงค์ (Indication)**	**ปริมาณ (Dose)**	**วิธีการ (Route)**	**ว/ด/ป ที่เริ่มใช้**

8. ใน 24 ชั่วโมงที่ผ่านมา วิธีการที่ท่านใช้บำบัดอาการปวดช่วยทำให้ท่านรู้สึกบรรเทาอาการปวดได้มากน้อยเพียงใด กรุณาประเมินและเลือกวงกลมลงบนตัวเลข

0 1 2 3 4 5 6 7 8 9 10

ไม่ช่วยบรรเทาอาการเลย บำบัดอาการปวดได้อย่างมีประสิทธิภาพที่สุด

9. กรุณาวงกลมลงบนตัวเลขเพื่อประเมินว่า ใน 24 ชั่วโมง ที่ผ่านมา อาการปวดนั้นได้รับ รบกวน การดำเนินชีวิตประจำวันของท่านในด้านต่างๆ มากน้อยแค่ไหน

8.1 กิจกรรมโดยทั่วไป

0 1 2 3 4 5 6 7 8 9 10

ไม่มีผลกระทบเลย มีผลกระทบอย่างมากที่สุด

8.2 อารมณ์

0 1 2 3 4 5 6 7 8 9 10

ไม่มีผลกระทบเลย มีผลกระทบอย่างมากที่สุด

8.3 ความสามารถในการเดิน

0 1 2 3 4 5 6 7 8 9 10

ไม่มีผลกระทบเลย มีผลกระทบอย่างมากที่สุด

8.4 งานประจำวัน (ทั้งงานประจำนอกบ้านและงานบ้าน)

0 1 2 3 4 5 6 7 8 9 10

ไม่มีผลกระทบเลย มีผลกระทบอย่างมากที่สุด

8.5 ความสัมพันธ์กับผู้อื่น

0 1 2 3 4 5 6 7 8 9 10

ไม่มีผลกระทบเลย มีผลกระทบอย่างมากที่สุด

8.6 การนอนหลับ

0 1 2 3 4 5 6 7 8 9 10

ไม่มีผลกระทบเลย มีผลกระทบอย่างมากที่สุด

8.7 ความสุขในการใช้ชีวิตประจำวัน

0 1 2 3 4 5 6 7 8 9 10

ไม่มีผลกระทบเลย มีผลกระทบอย่างมากที่สุด

Pain diagnosis by ICD11 and pain grouping into2, 3 or 4

1________________ (group______)

2________________ (group______)

3________________ (group______)

If the patients have pain that belong to group 2, schedule for the next (2nd or 3rd) interview (3 and 9 months after the first interview)

Interview2 Date ☐☐/☐☐/☐☐☐☐ by_______________

Interveiw3 Date ☐☐/☐☐/☐☐☐☐ by_______________
