# Supplementary material for: The incidence, characteristics, impact and risk factors of post-COVID chronic pain in Thailand: A single-center cross-sectional study
Source: PLoS One. 2024 Jan 12;19(1):e0296700. doi: 10.1371/journal.pone.0296700 (PMC10786369; doi:10.1371/journal.pone.0296700)
Supplement: S3 Appendix — (DOCX) [file pone.0296700.s005.docx]

**S3 Appendix**

**Table** type of vaccine before infection

| Type of vaccine before infection, n (%) | (n=1,019) |
| --- | --- |
| No vaccine | 340 (33.4) |
| Inactivated virus (Sinovac, Sinopharm) | 251 (24.6) |
| Viral vector (AstraZeneca) | 416 (40.8) |
| mRNA (Pfizer, Moderna) | 12 (1.2) |

**Table** Comparison vaccine type between Group 1 (no pain), Group 2 (post-COVID chronic pain, PCCP), Group 3 (chronic pain with aggravated pain), and Group 4 (chronic pain without aggravated pain).

| Total sample  (n = 1,019) | Group 1  (n = 880) | Group 2  (n = 33) | Group 3  (n = 38) | Group 4  (n = 68) | p-value |
| --- | --- | --- | --- | --- | --- |
| Type of vaccine before infection, n (%) |  |  |  |  | 0.024^a^ |
| No vaccine | 279 (31.7) | 9 (27.3) | 19 (50.0) | 33 (48.5) |  |
| Death virus (Sinovac, Sinopharm) | 228 (25.9) | 10 (30.3) | 6 (15.8) | 7 (10.3) |  |
| Viral vector (AstraZeneca) | 361 (41.0) | 14 (42.4) | 13 (34.2) | 28 (41.2) |  |
| mRNA (Pfizer, Moderna) | 12 (1.4) | 0 (0.0) | 0 (0.0) | 0 (0.0) |  |

^a^Statistically significant relative risk.

Table Relative risk of vaccine type of post-COVID chronic pain (PCCP, Group 2) with those individuals not developing pain (Group 1).

|  | Group 1  (n = 880) | Group 2  (n = 33) | p-value | RR (95%CI) |
| --- | --- | --- | --- | --- |
| Type of vaccine before infection, n (%) |  |  | 0.024 | - |
| No vaccine | 279 (31.7) | 9 (27.3) | - | - |
| Inactivated virus (Sinovac, Sinopharm) | 228 (25.9) | 10 (30.3) | - | 0.9 (0.5-1.4) |
| Viral vector (AstraZeneca) | 361 (41.0) | 14 (42.4) | - | 0.7 (0.4-1.2) |
| mRNA (Pfizer, Moderna) | 12 (1.4) | 0 (0.0) |  | 1.0 (1.0-1.1) |

^a^Statistically significant relative risk.

**Table** Relative risk of vaccine type among chronic pain with aggravated pain after COVID-19 (Group 3) in relation to chronic pain without aggravated pain.

|  | Group 3  (n = 38) | Group 4  (n=68) | p-value | RR (95%CI) |
| --- | --- | --- | --- | --- |
| Type of vaccine before infection, n (%) |  |  | 0.632 | - |
| No vaccine | 19 (50.0) | 33 (48.5) | - | - |
| Inactivated virus (Sinovac, Sinopharm) | 6 (15.8) | 7 (10.3) | - | 1.4 (0.5-3.6) |
| Viral vector (AstraZenaca) | 13 (34.2) | 28 (41.2) | - | 0.9 (0.5-1.5) |
| mRNA (Pfizer, Moderna) | 0 (0.0) | 0 (0.0) | - | - |

**Table** demonstrated pain interferences among groups 2-4

| BPI  (n = 139) | Group 2  (n = 33) | Group 3  (n = 38) | Group 4  (n = 68) | P value |
| --- | --- | --- | --- | --- |
| Pain interferences |  |  |  |  |
| Activity | 2.4 ± 2.6 | 3.0 ± 3.1 | 3.0 ± 3.1 | 0.521 |
| Mood | 2.4 ± 2.3 | 2.6 ± 3.1 | 2.8 ± 2.8 | 0.784 |
| Mobility | 2.5 ± 3.0 | 3.5 ± 3.3 | 3.6 ± 3.2 | 0.264 |
| Work | 1.7 ± 2.6 | 3.3 ± 3.2 | 2.8 ± 3.2 | 0.080 |
| Relationship | 1.4 ± 2.5 | 1.5 ± 2.8 | 1.0 ± 2.1 | 0.586 |
| Sleep | 2.2 ± 2.8 | 3.6 ± 3.3 | 2.2 ± 3.1 | 0.053 |
| Happiness | 2.4 ±2.5 | 2.5 ± 2.7 | 2.3 ± 2.5 | 0.957 |
